# A systematic review of the relationship between race and health-related quality of life outcomes in patients with advanced heart failure who undergo heart transplantation or mechanical circulatory support

**DOI:** 10.1007/s11136-026-04208-w

**Published:** 2026-04-01

**Authors:** Jerian Dixon-Evans, Alexis Briley, Jamie S. Way, Shondra Clay, Kathryn Mazurek, Q. Eileen Wafford, Alyssa M. Vela, Kathleen L. Grady

**Affiliations:** 1 Department of Surgery, Division of Cardiac Surgery, Northwestern University, Feinberg School of Medicine, 676 North Saint Clair Street, Arkes Pavilion, Suite 730, Chicago, IL 60611-3056 USA; 2 College of Health and Human Sciences, Northern Illinois University, Wirtz Hall, 323, DeKalb, IL 60115 USA; 3 Department of Physical Therapy and Health Science, Bradley University, Campustown 170, 1501 W. Bradley Ave., Peoria, IL 61625 USA; 4 Feinberg School of Medicine, Northwestern University, 320 E. Superior Street, Chicago, IL 60611 USA

**Keywords:** Heart transplantation, Left ventricular assist device, Health-related quality of life, Health equity, Race, Mechanical circulatory support

## Abstract

**Purpose:**

Health inequalities may disproportionately affect health-related quality of life (HRQOL) in patients with heart failure who identify as a racial or ethnic minority and undergo advanced surgical therapies. Objectives of this systematic review were to determine the impact of race on HRQOL of adults who undergo heart transplantation (HT) or durable mechanical circulatory support (MCS), while awaiting HT or long-term, and whether race is a risk factor for worse HRQOL.

**Methods:**

A synthesis framework was used following the Preferred Reporting Items for Systematic Reviews and Meta-Analyses 2020 guidelines. Medical Subject Headings was applied to search engines. Peer reviewed articles (English) published prior to 1/5/25, examining associations between race & HRQOL of adults who underwent HT and/or MCS, were included. Excluded were systematic reviews, editorials/opinions, case series/reports, grey literature, qualitative research, and case–control studies. Risk of bias was assessed using Joanna Briggs Institute checklists and the Cochrane Risk-of-Bias tool.

**Results:**

Among 2,902 identified records, eight studies met inclusion criteria (HT: n = 4; MCS: n = 3; and HT and MCS: n = 1). Participants’ mean age = 57 years; the majority were White (80%) and male (78%). Risk of bias varied by study. Race did not impact HRQOL in 50% of HT studies. HRQOL improved with MCS in all studies; however, compared to White patients, Black patients experienced less improvement in HRQOL.

**Conclusions:**

HRQOL measures were not comparable. Thus, a meta-analysis was not performed which is a study limitation. Racial differences in HRQOL may exist among adults who undergo HT and/or MCS; more research is needed.

**Supplementary Information:**

The online version contains supplementary material available at 10.1007/s11136-026-04208-w.

## Introduction

Researchers have documented a disproportionate burden of heart failure borne by racial and ethnic minorities and worse outcomes, such as premature deaths and increased rates of hospital readmission [1]. In the United States (U.S.), over 7.5 million adults suffer from heart failure, and the incidence and prevalence increases with age [2, 3]. Heart failure occurs when the heart muscle is unable to pump enough blood and oxygen to meet the body’s needs [4]. Nearly 10% of patients with heart failure have progressed to end-stage disease (i.e., advanced heart failure) [4]. There has been significant progress in the surgical treatment (i.e., heart transplantation [HT] and durable mechanical circulatory support [MCS]) of advanced heart failure over the last 20 years, with increasing rates of survival and enhanced health-related quality of life (HRQOL) [5–7]. Despite the benefits of these advanced cardiac surgical therapies, racial and ethnic inequalities exist among patients with advanced heart failure [7]. Notably, Black males and females experience higher mortality rates than their White counterparts [8]. Specifically, heart failure death rates (per 100,000) are highest for Black males (123.3) and Black females (88.9) when compared with White males (115.3) and White females (82.7) [2]. Blacks and Hispanics are also more likely to develop heart failure at a younger age than White individuals [9, 10].

Structural racism and systemic barriers contribute to health disparities in patients who undergo advanced cardiac surgical therapies such as implantation of left ventricular assist devices (LVADs), a type of MCS, and HT. Black patients have less access to these therapies despite having higher rates of heart failure compared to White patients [11]. Black patients are 10% less likely to receive a transplant, and they experience a 14% higher risk of mortality post-operatively [11]. Hispanics have a 50% higher risk of death while on the HT waiting list, and Blacks have a higher risk of organ rejection and mortality after transplantation compared to White patients (Blacks: 57.9% versus Whites: 37.8%) [12–14]. These disparities are driven by a complex interaction of issues, including provider bias, structural inequities, and social determinants of health, which all influence clinician decision-making and referral patterns [15].

There is a paucity of knowledge regarding the effect of race on HRQOL outcomes for patients with advanced heart failure who undergo HT or durable MCS, either while awaiting HT or if ineligible for HT, long-term. Therefore, we conducted a systematic review addressing the following research question: What is the relationship between race and HRQOL of adult patients (age 18 + years) with heart failure who undergo advanced cardiac surgical therapies (i.e., MCS or HT)? This systematic review will identify literature addressing this question and provide guidance for future research examining differences in HRQOL by race, which may ultimately guide shared decision making, as patients with advanced heart failure consider surgical therapeutic options. The objectives of this systematic review are to determine (1) the impact of race on HRQOL of adult patients who undergo HT or durable MCS while awaiting HT or if ineligible, long-term, and (2) if race is a risk factor for worse HRQOL outcomes.

## Methods

The Preferred Reporting Items for Systematic Reviews and Meta-Analyses (PRISMA) 2020 statement was used to conduct this systematic review [16]. The protocol was registered with The International Prospective Register of Systematic Reviews (PROSPERO) (ID-CRD42021266728; https://www.crd.york.ac.uk/PROSPERO/view/CRD42021266728).

### Search strategy

A research librarian (QEW) was consulted to develop a comprehensive database search strategy. Full search strategies are available in Supplemental Tables S1-S7. The search strategy combined keywords and Medical Subject Headings (MeSH) describing adults, MCS, HT, and U.S. racial and ethnic minorities as identified by the American Community Survey, 1-Year Estimates Data Profiles [17]. The search was applied to Medline (Ovid), The Cochrane Library (Wiley), Scopus (Elsevier), CINAHL Plus with Full Text (EBSCOhost), and PsycInfo (EBSCOhost) on June 5, 2025. We searched ClinicalTrials.gov for additional studies. The reference lists of included studies were also examined for relevant citations.

### Eligibility criteria

Articles were evaluated for eligibility (Table 1) and included if they met the following criteria: (a) peer-reviewed articles; (b) English language published before June 5, 2025; and (c) original research conducted in the U.S. investigating the relationship between race and HRQOL among adult patients diagnosed with advanced heart failure who underwent either HT or MCS, (specifically a durable LVAD). Additionally the following publication types (meta-analysis, systematic reviews, editorials/opinions, case series/reports and grey literature [e.g., theses, dissertations, conference proceedings, and abstracts]) and research methodologies (qualitative research, case control studies) were excluded from this review. This was done primarily to avoid duplicating data, reduce risk of bias, improve evidence certainty, and maintain methodological consistency across included studies.

**Table 1:** Overview of the criteria for inclusion and exclusion of articles in the review

Inclusion criteria	Exclusion criteria
Study designs will be included based on the criteria: Observational Cohort Study Quantitative Research Randomized Controlled Trial	Publication types: Grey literature (theses, dissertations, conference proceedings, and abstracts) Meta-analysis Systematic reviews Editorials/opinions pieces Case series/reports
Adults: ≥ 18 y Advanced heart failure diagnosis	Qualitative Studies Case Control Studies
U.S. studies	Studies that did not report on both race and HRQOL^1^ outcomes
People who have received a heart transplant or mechanical circulatory support device, either while awaiting heart transplantation or long-term	Advanced heart failure studies whose research participants did not receive either a heart transplant or long-term mechanical assist device (specifically the left ventricular assist device [LVAD])
Publications wherein racial and/or ethnic demographic information was collected American Indian or Alaskan Native Asian Black or African American Hispanic or Latino Native Hawaiian or other Pacific Islander White	
Publications that examine HRQOL^1^	

^1^*HRQOL* health-related quality of life

### Study selection

**Fig. 1:**
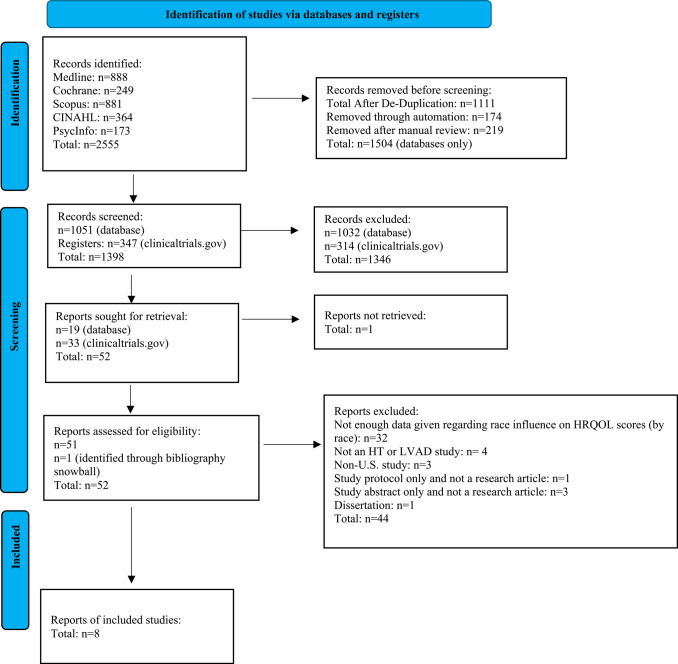
PRISMA Flow Diagram. *Note*. Includes searches of databases, registers, and other sources. Adapted from “Preferred Reporting Items for Systematic Reviews and Meta-Analyses: The PRISMA Statement 2020,” by Page et al. (2021)

The review team consisted of three researchers: JDE (lead), JW and AB. Studies were independently evaluated through review of the study title and abstract to determine relevancy and potential for inclusion. All papers identified in the search strategy were uploaded for screening after de-duplication in Rayyan. Relevant articles meeting eligibility criteria were investigated further by evaluation of the full text; discrepancies regarding inclusion of articles were resolved through consensus. If there was no resolution, the content expert, KG, was consulted to review the article for final inclusion. The search retrieved 1,051 unique articles and 347 clinical trial records as shown on the PRISMA-P Flow Diagram, 2020 version (Fig. 1). A total of eight articles that met inclusion criteria were included in this review.

### Data collection

To extract information from studies, three reviewers (JDE, AB, and JW) used a data extraction excel workbook. They independently collected details such as participant characteristics (e.g., age, gender, race/ethnicity, and heart failure severity using the New York Heart Association [NYHA] class), study methodology, intervention details relevant to the study design, HRQOL measures, findings, and study limitations. Articles were identified by title, authors, journal name, and publication year.

### Data analyses

Demographics, clinical variables, and HRQOL measures were summarized using means and standard deviation, or counts and percentage, as appropriate. Critical appraisal analyses were conducted using tools to address risk of bias, as appropriate to the study design. The risk of bias for each included study was analyzed by three reviewers (AB, AV, and JW). We used a tool developed by the Cochrane Collaboration risk-of-bias (RoB 2) to evaluate risk of bias in randomized trials [18]. The RoB 2 provides insight into five possible areas that may introduce bias into randomized trials results [18]. Using the risk-of-bias grading system, each outcome criterion is categorized as low risk of bias, some concerns, or high risk of bias [18]. For prevalence and cohort studies, we used the Joanna Briggs Institute (JBI) Critical Appraisal Checklist. The responses for both checklists are: Yes, No, Unclear, or Not Applicable [19, 20].

### Data synthesis

A narrative synthesis framework was used to synthesize data. In narrative synthesis, the results of multiple studies are synthesized using words which may be supported by statistical evidence [21]. It was not appropriate to apply a meta-analysis approach for this review due to the wide range of HRQOL measures used in the articles [22, 23].

## Results

### Demographic and clinical characteristics

Of the eight studies included in this review, four focused on HT [25–27, 30], three focused on MCS [24, 28, 31] (both while awaiting HT and long-term MCS) and one included both HT and MCS [29] (Table 2). The sample sizes for these studies ranged from *n* = 47 to *n* = 960. Six of the eight studies had diverse study populations reported as White, Black, Hispanic, Asian or other [24–29]. Two studies only reported race as either Black or White [30, 31].

**Table 2:** Summary of study characteristics, quality of life measures, and main findings of articles included in the review

1st author (year)	Study design	Intervention details	Sample size	Age (mean ± *SD*)	Gender	Race	New York Heart Association (NYHA) class	HRQOL/QOL measures	Findings
Allen (2010) [28]	Cohort	Aim: To evaluate QoL and Functional Status measures Population: Patients implanted with LVAD ≥ 1 year Patients were implanted with either a HeartMate pulsatile (HMl) or continuous-flow (HM II) LVADs from 2000 to 2009	*n* = 103	1-year survivors: 48.2 (± 12.4) years Died in 1st year: 49.8 (± 13.7) years	1-year survivors: Male: 73.3% Female: 26.7% Died in 1st year: Male: 79.4% Female: 20.6%	1-year survivors: White: 40% Black: 56.7% Hispanic: 3.3% Died in 1st year: White: 61.6% Black: 31.5% Hispanic: 4.1%	93% were NYHA Class IV at baseline	MLHFQ	-MLHFQ scores of Black 1-year survivors were lower (better) than White 1-year survivors -LVAD patients spent more time outside the hospital and have higher QoL scores -No difference in QoL indicators when stratified by LVAD type
Barr (2003) [25]	Cross-sectional; Longitudinal Cohort	Aim: To assess the QOL of up to 1-year, stable survivors (surviving ≥ 5 Y) after heart transplantation Cross-sectional and longitudinal multivariate analyses were used to investigate: (1) what characteristics identify individuals with higher (or lower) QOL scores than other, and (2) which characteristics influence changes in QOL for an individual during a 1-year period? Data analyzed using the LSI and the TCI	*n* = 569	61.7 (8.8) years	Male: 76% Female: 24%	White: 87% Black: 5% Hispanic: 2% Asian: 1% Other: 5%	Data not included for NYHA	LSI	-Being married was correlated with increased QOL -Higher QOL scores were seen in patients who were older -QOL decreased in the LSI scores for patients with more comorbid conditions as compared to those with fewer comorbid conditions
Dixon-Evans (2025) [29]	Cohort	Aim: To determine whether (1) older patients (60–80 years) with HF who underwent long-term MCS, compared to patients who underwent HT, with (HT MCS) or without (HT non-MCS) pretransplant MCS, experienced noninferior change in overall HRQOL by race (White vs racial minorities) from baseline to 1-year postoperatively and (2) race was a risk factor associated with overall HRQOL at 1-year postoperatively MCS devices included second or third generation Food and Drug Administration-approved or investigational durable continuous flow left ventricular assist devices (LVADs, HeartMate II, HeartMate 3, and HeartWare)	*n* = *396*	66 ± 4.7 years	Male: 78% Female: 22%	White: 83% Black: 14% Asian: 2% Hawaiian or Pacific Islander: 1%	NYHA IV (65% of patients), NYHA III (19%), and NYHA II (13%) (baseline)	KCCQ-12	-In the multivariable analysis, sex (male), and surgery strategy (HT MCS, and HT non-MCS) were positively associated with the KCCQ-12 OSS, while the cumulative number of postoperative adverse events was negatively associated with KCCQ-12 OSS -Clinically important differences in overall HRQOL by race were observed, with White patients reporting higher KCCQ-12 OSS than racial minorities -Race was found not to be a risk factor for poorer HRQOL outcomes using the KCCQ-12 OSS
Evangelista (2004) [26]	Cross-sectional	Aim: To identify and evaluate the QOL and psychological well being of transplant candidates and recipients To detect factors that are associated with improved QOL among female recipients of heart transplants through questionnaires	*n* = 100	Transplant recipients: 54.7 ± 13.0 years Transplant candidates: 56.8 ± 12.2 years	All participants were female; Candidates: 50% Recipients:50%	Recipients: White: 70% Black: 12%, Other: 18% Candidates: White: 74% Black: 14% Other: 12%	Transplant candidates: (baseline) NYHA Class II, 13% NYHA Class III, 53.7% NYHA Class IV, 33.3% Transplant recipients: NYHA Class I, 70% NYHA Class II, 30%	MLHFQ, BDI, and CAS	-Lower (better) QOL scores for female heart transplant recipients -Both groups reported poor QOL (emotional distress) and moderate-to-severe depression -Functional status in older participants was lower, while the physical health status score was higher (poorer) -No association between sociodemographic characteristics (including race, marital status, education, employment status, and income) and QOL or psychological well-being scores among transplant recipients
Grady (2002) [24]	Observational, Cross-sectional	Aim: to describe QOL outcomes, ascertain the relationship between QOL and demographic, physical, psychosocial, and clinical variables, and characterize predictors of QOL at 1 month post − LVAD surgery Patients were implanted with either an implantable pneumatic or a vented electric HeartMate LVAD	implantable pneumatic (*n* = 38) vented electric (*n* = 54) Heart Mate LVAD as a BTT	51.5 ± 11.7 years	Male: 92%, Female: 8%	White: 79% Black: 17% Other: 2%, Hispanic 1%	NYHA IV (94% of patients), NYHA III (4%), and NYHA II (2%) (baseline)	QLI;RQF;HFSC;SIP; LVAD SS; JCS	-Some QOL improvement seen 1 month post-implantation -High level of satisfaction with QOL and post-surgery outcomes 1 month after LVAD implant -Black participants reported fewer psychological difficulties and were more satisfied with their overall QOL 1 month post − LVAD surgery
Grady (2005) [27]	Observational, cohort study	Aim: to describe QOL, identify predictors of QOL at 5 to 6 years post-transplantation and identify changes in QOL by age, gender, and race Study participants self-reported their QOL on 12 questionnaires using multiple measures of QOL and other outcomes	*n* = 231	59.0 ± 10.3 years	Male: 76% Female: 26%	White: 90% Black: 7% Other: 3%	Data not included for NYHA	HTSC; HTSS; JCS; SIP; QoLI; THIS;SSI;APTR; PNAS; CDS	-Patients aged 60 years and older reported being more satisfied with their QOL in comparison to their younger counterparts –Nearly 80% of the difference in satisfaction with QOL was associated with psychological, physical, social, clinical, and demographic factors at 5–6 years following heart transplantation -Race was the only factor that was associated with a difference in satisfaction with QOL -White heart transplant recipients reported higher levels of satisfaction with their socio-economic status than non-White recipients
Salyer (2001) [30]	Pilot Study; cross-sectional, descriptive study	Aim: to explore the lifestyle and health status of long-term cardiac transplant recipients over time	*n* = 47	Males: 57.19 (23.13) years Females: 54.27 (23.13) years	Male: 77% Female: 23%	White: 72% Black: 28%	Data not included for NYHA	Health-Promoting Lifestyle Profile II	- There was no significant difference in health status between White and Black participants -Findings suggested poorer stress management behaviors among Black people may contributing to elevated BP levels -Black participants reported that their stress management was poorer than White participants
Sheikh (2021) [31]	Prospective, multicenter, randomized clinical trial	Aim: To assess the safety and efficacy of the HeartMate 3 LVAD by demonstrating non-inferiority to the HeartMate II LVAD, for short-term and long-term support	*n* = 960* *1015 patients enrolled in the study; *n* = 55 self-identified as Asian, Hispanic/ Latino, Native Hawaiian or other Pacific Islander were excluded from the analysis	White: 62.3 ± 11.4 years Black: 53.9 ± 12.1 years	Male: 79% Female: 21%	White: 70% Black: 30%	Data not included for NYHA INTERMACS profile > 3: White: 18.8% Black: 7.1%	EuroQol group, 5-dimension, 5-level instrument visual analog scale (EQ-5D-VAS)	-At 2 years, Black and White HM3 recipients had equivalent survival rates free from disabling strokes or the need for surgery to remove or replace malfunctioning devices -Hospitalization and length of stay rates were higher for Black participants than White participants -Compared to White participants, Black Heart Mate 3 participants had greater morbidity burden and achieved less improvement in functional capacity and the degree of change in the QoL measurement from baseline to 6 months was lower post-implantation

*QoL* Quality of Life, *LVAD* Left Ventricular Device, *NYHA* New York Heart Association, *MLHFQ* Minnesota Living with Heart Failure Questionnaire, *LSI* Life Satisfaction Index, *TCI* Transplant Care Index, *BDI* Beck Depression Inventory, *CAS* Control Attitude Scale, *HM3* Heart Mate 3, *LVAS* Left Ventricle Assist System, *EQ-5D-VAS* EuroQol group, *5 dimension* 5 level instrument visual analog scale, *QLI* Quality of Life Index, *RQF* Rating Question Form, *HFSC* Heart Failure Symptom Checklist, *SIP* Sickness Impact Profile, *LVAD SS* LVAD Stressor Scale, *JCS* Jalowiec Coping Scale, *HTSC* Heart Transplant Symptom Checklist, *HTSS* Heart Transplant Stressor Scale, *QoLI* Quality of Life Index, *HTIS* Heart Transplant Intervention Scale, *SSI* Social Support Index, *APTR* Assessment of Problems With Transplant Regimen, *PNAS* Positive and Negative Affect Schedule, *CDS* Cardiac Depression Scale, *INTERMACS* Interagency Registry for Mechanically Assisted Circulatory Support., *KCCQ-12* Kansas City Cardiomyopathy Questionnaire 12

Participants in the studies were, on average, 57 years of age, with a range = 48–69 years. The majority were White (range = 40%-90%) and male (range = 73–92%). Of note, the study conducted by Evangelista et al. (2004) included an all-female sample [26]. Marital status was also reported in six of the included studies with the majority of participants being in a spousal relationship regardless of surgical strategy, HT or MCS [24–27, 29, 30]. Five of the studies reported education; the majority of participants had at least a high school education [24–26, 29, 31]. Four studies did not include data on NYHA class; of the four studies that reported this information, most participants were NYHA Class III and IV [24, 26, 28, 29]. Five studies provided an etiology of heart failure, most commonly ischemic cardiomyopathy [27–31].

### Health-related quality of life measures

The most commonly used HRQOL measures were the Minnesota Living With Heart Failure Questionnaire (MLHFQ; *n* = 2) [26, 28] and the Quality of life Index (QLI; *n* = 2) [24, 27]. Additional HRQOL measures used in these studies included the EuroQol 5-Dimension 3-Level (EQ-5D-3L); (*n* = 1) [31], Health-Promoting Lifestyle Profile II (*n* = 1) [30], Life Satisfaction Index (LSI; *n* = 1) [26], and Kansas City Cardiomyopathy Questionnaire-12 (KCCQ-12) (*n* = 1) [29]. All of these HRQOL measures have domains/subscales except the LSI. Some studies used additional measures that included specific HRQOL domains (e.g., Sickness Impact Profile, Positive and Negative Affect Schedule, and Cardiac Depression Scale [*n* = 2]) [24, 27]. Additional details about these measures are found in Supplemental Table 1.

### Study design

Study designs of the eight articles are presented in Table 2. This systematic review included four cross-sectional/prevalence studies [24–26, 30], one randomized controlled trial [31], and four cohort studies [25, 27–29]. The study by Barr et al. (2003) had both a cross-sectional and a cohort design [25].

### Critical appraisal analyses

Using the JBI Critical Appraisal Checklists to determine risk of bias in the prevalence and cohort studies [24–30], we summarized our assessments to determine if authors of studies have taken steps to minimize bias in their study design, conduct, and analyses (Tables 3 and 4). The majority of studies were assessed as having a low risk of bias; however, a few received an 'unclear' or ‘not applicable’ rating on specific items of the questionnaire. Based on the reviewers’ appraisal, the studies conducted by Grady et al. (2002), and Evangelista et al. (2004) were rated as ‘unclear’ with respect to participant sampling methods due to insufficient reporting (see Table 3) [24, 26]. Similarly, Salyer et al. (2001), used a small convenience sample and was assigned an ‘unclear’ rating on the JBI risk of bias questionnaire regarding adequacy of the sample size (Table 3) [30]. Other concerns of potential bias were noted in the study by Allen et al. (2010) whose study was retrospectively sampled. The primary outcome in the studies conducted by Allen et al. (2010), Grady et al. (2005), and Dixon-Evans et al. (2025) was HRQOL. When evaluating these studies using the JBI checklist for cohort studies, the question of whether the sample was free of the outcome at the start of the study (or exposure) was marked as ‘not applicable’ by the reviewers as individuals cannot be ‘free’ of HRQOL. The review team independently assessed the study by Sheikh et al. (2021) using the RoB 2 and determined it to have a low risk of bias (Table 5) [31].

**Table 3:** Joanna Briggs institute critical appraisal checklist applied for included studies reporting incidence and prevalence data

1st author (year)	Was the sample frame appropriate to address the target population?	Were study participants sampled in an appropriate way?	Was the sample size adequate?	Were the study subjects and the setting described in detail?	Was the data analysis conducted with sufficient coverage of the identified sample?	Were valid methods used for the identification of the condition?	Was the condition measured in a standard, reliable way for all participants?	Was there appropriate statistical analysis?	Was the response rate adequate, and if not, was the low response rate managed appropriately?
Barr (2003)	Yes	Yes	Yes	Yes	Yes	Yes	Yes	Yes	Yes
Evangelista (2004)	Yes	Unclear	Yes	Yes	Yes	Yes	Yes	Yes	Yes
Grady (2002)	Yes	Unclear	Yes	Yes	Yes	Yes	Yes	Yes	Yes
Salyer (2001)	Yes	Yes	Unclear	Yes	Yes	Yes	Yes	Yes	Yes

Prevalence studies critical appraisal tool. Answers: Yes, No, Unclear, or Not/Applicable

Source: Evangelista et al., 2004. Authors noted limitations imposed by the review board, only those participants who expressed interest in participating in the study were screened and enrolled. Grady et al., 2002. Authors noted that the quality of life at 1 month may have been overestimated due to half of the patients being too sick to complete the questionnaires. Salyer et al., 2001. Authors noted in the article that they used a small convenience sample which may have been a study limitation. There may be other important relationships that were overlooked in this pilot study as a result of being underpowered

**Table 4:** Joanna Briggs institute critical appraisal checklist applied for included cohort studies

1st author (y)	Were the two groups similar and recruited from the same population?	Were the exposures measured similarly to assign people to both exposed and unexposed groups?	Was the exposure measured in a valid and reliable way?	Were confounding factors identified?	Were strategies to deal with confounding factors stated?	Were the groups/participants free of the outcome at the start of the study (or at the moment of exposure)?	Were the outcomes measured in a valid and reliable way?	Was the follow-up time reported and sufficient to be long enough for outcomes to occur?	Was follow-up complete, and if not, were the reasons to loss to follow-up described and explored?	Were strategies to address incomplete follow-up utilized?	Was appropriate statistical analysis used?
Allen (2010)	Not/ Applicable	Yes	Yes	Yes	Yes	Yes	Yes	Yes	Unclear	Unclear	Yes
Barr (2003)	Yes	Yes	Yes	Yes	Yes	Not/Applicable	Yes	Yes	Yes	Yes	Yes
Grady (2005)	Yes	Yes	Yes	Yes	Yes	Not/Applicable	Yes	Yes	Yes	Yes	Yes
Dixon-Evans (2025)	Yes	Yes	Yes	Yes	Yes	Not/Applicable	Yes	Yes	Yes	Yes	Yes

Cohort studies critical appraisal tool. Answers: Yes, No, Unclear, or Not/Applicable

Allen et al., 2010. Authors note that they retrospectively reviewed their prospective database to identify patients with axial-flow HeartMate or pulsatile-flow HeartMate LVADs. As the outcome of this study was QOL, leading to the designation of Q#6 as not applicable. QOL is not a variable that ‘starts’ at any given point or in response to an intervention

Grady et al., 2005. As the outcome of this study was QOL, leading to the designation of Q#6 as not applicable. QOL is not a variable that ‘starts’ at any given point or in response to an intervention

Dixon-Evans et al., 2025. As the outcome of this study was QOL, leading to the designation of Q#6 as not applicable. QOL is not a variable that ‘starts’ at any given point or in response to an intervention

**Table 5:**
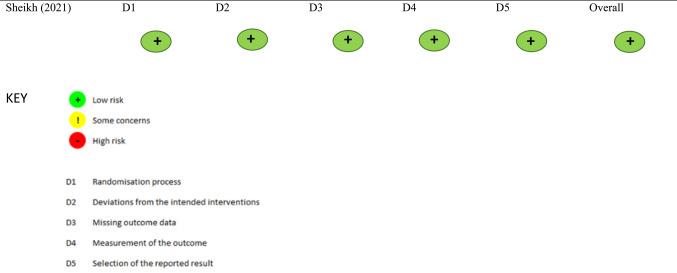
Revised Cochrane Risk-of-Bias Tool for Randomized Trials (RoB 2)

### HT and MCS study findings

Among the four HT studies examined, 50% found no significant relationship between race and HRQOL, nor any association with psychological well-being [25, 26]. Additionally, Dixon-Evans et al., (2025) found no significant differences at baseline by race in the KCCQ-12 overall summary score (OSS) within two HT groups (with and without pre-transplant MCS) [29]. However, their findings did reveal a clinically important (> 5-point) difference (as determined by the authors of the KCCQ-12) [32] in the HT without prior MCS group by race, wherein White patients had a higher (i.e., better health status) KCCQ-12 OSS than racial minorities, at 3- and 6- months follow-up which became statistically significant at 1-year follow-up [29].

Additionally regarding domains, Grady et al. (2005) reported that White HT recipients expressed greater satisfaction with their socioeconomic status in comparison to non-White recipients [27]. Also, older HT recipients (over 60 years of age) were more satisfied with their HRQOL than younger recipients [27]. Furthermore, Salyer et al. (2001) indicated that Black HT recipients demonstrated less effective stress management behaviors than their White counterparts [30]. In female HT recipients, even though they had lower (better) MLHFQ scores, 25% experienced moderate-to-severe depression [26].

Regarding MCS studies, improvement in HRQOL was reported by all patients, regardless of surgical intent (while awaiting HT or long-term). Specifically, Black patients demonstrated high level of satisfaction with HRQOL at 1 month post-implantation [24] and had lower (i.e., better) MLHFQ scores at 1 year follow-up [28]. A meaningful finding was that while Black patients experienced improvement in HRQOL, in comparison to White patients, improvement was less pronounced and represented a non-significant trend [31]. Furthermore, in the Dixon-Evans et al., (2025) study, they found that despite improvement in all participants’ KCCQ-12 OSS after 1 year post-surgery, there was a strong trend for White patients to have higher HRQOL scores than racial minorities [29]. Additionally, Allen et al., (2010) found that when stratified by MCS device type (HeartMate 1® versus HeartMate 2®), HRQOL did not differ by group [28].

## Discussion

To our knowledge, this systematic review is the first to examine the relationship of race and HRQOL in patients with heart failure who underwent advanced cardiac surgical therapies (i.e., HT and/or MCS). After applying inclusion criteria, eight articles were analyzed (four HT studies, three MCS studies, and one HT and MCS study). Based on this review, definitive conclusions about whether race is an independent risk factor for poor HRQOL outcomes cannot be determined given the limited and heterogenous nature of the literature. Generalization to a larger population of racially diverse patients with advanced heart failure who undergo these surgical therapies cannot be made. Thus, there are clear gaps in knowledge of whether and how HRQOL is impacted by race for patients who undergo HT or MCS implantation.

Race has been previously investigated as a risk factor primarily in HT and MCS patients studied separately. Findings from the Multi-center Study of MagLev Technology in Patients Undergoing Mechanical Circulatory Support Therapy with HeartMate 3 (MOMENTUM 3) trial indicated that after adjusting for age there was no significant impact of sex, race, severity of illness, and therapeutic intent on primary end-points (i.e., survival free of disabling stroke and reoperation to replace or remove the device) [33]. Given the paucity of literature investigating the intersection of race and HRQOL outcomes among patients undergoing HT or MCS, findings from the broader heart failure literature provide additional insights for interpreting patterns observed in this systematic review for patients who undergo advanced cardiac surgical therapies. In the observational study, Change the Management of Patients with Heart Failure (CHAMP-HF), investigators reported that females, Blacks, Hispanics, and patients from lower socioeconomic backgrounds had a worse overall mean KCCQ OSS in comparison to males and White patients [34]. Collectively, these findings demonstrate inconsistent associations between race and clinical and patient-reported outcomes.

Findings related to HRQOL across heart failure literature remain mixed. Some studies, such as the Rehabilitation Therapy in Older Acute Heart Failure Patients (REHAB-HF) trial, reported no significant difference in the KCCQ OSS and physical domain scores between Black and White patients, with both groups experiencing improvement in their scores [35]. In contrast, other studies, including the Palliative Care in Heart Failure Trial, revealed that Black patients had higher mean KCCQ scores at baseline compared to White patients and although not statistically significant, improvement in scores was greater among Black patients [36]. Similar patterns were reported in the Registry Evaluation of Vital Information for VADs in Ambulatory Life (REVIVAL) study, wherein Black patients had a higher, but non-significant baseline KCCQ OSS and EQ-5D-3L visual analogue scale (VAS) score than their White counterparts [37]. These contrasting results mirror the patterns observed in the present systematic review, in which no consistent relationship between race and HRQOL emerged among patients undergoing HT or MCS.

Notably, the number of racially diverse patients with advanced heart failure who undergo these therapies is increasing. Thus, there is a clear need for additional research to evaluate the effects of race on HRQOL for patients of diverse racial and ethnic backgrounds who have heart failure, including those who undergo HT and LVAD implantation. Data from the United Network for Organ Sharing registry indicated that the number of Black individuals listed for HT increased from 21.7% to 28.2% between 2011 to 2020 [38]. Additionally from 2012 to 2015, the rate of VAD implants among Black patients increased by 0.26 per 100,000 [39]. Using the Interagency Registry for Mechanically Assisted Circulatory Support (Intermacs) and Medicare data, Bourque et al. (2019) reported an increase from 2008 to 2014 in use of durable MCS devices among women (20.04% to 22.75%) and racial and ethnic minority populations (29.7% to 32.3%) [40]. Although use has increased, disparities still exist and may influence postoperative HRQOL for patients undergoing HT or MCS procedures.

Despite the lack of consistent racial differences in HRQOL identified in this systematic review, health disparities persist in advanced heart failure and may be driven, in part by structural racism [41]. Black patients undergoing advanced surgical therapies remain less likely to receive MCS devices or HT, even after adjusting for disease severity, HRQOL, and social determinants of health [42, 43]. These findings suggest the influence of provider bias, systemic barriers, and discriminatory practices on transplant evaluation and allocation processes [44, 45]. There is a need for public policy reform, provider bias training, and expanded access to advanced cardiac therapies, which may improve health outcomes, including HRQOL, for all patients, regardless of race and ethnicity [46]. Although conclusive findings could not be drawn, this systematic review has identified key gaps in the literature and establishes essential groundwork for future studies to examine the impact of race on HRQOL after advanced cardiac surgical therapies (i.e., HT or MCS).

Limitations were identified in this systematic review. This review included only U.S. studies; inclusion of studies from other countries may have enhanced racial and ethnic diversity and been more generalizable. A significant limitation of the present systematic review lies in the non-comparability of HRQOL measures. Consequently, the use of a meta-analysis approach was deemed unsuitable. Additionally, there was considerable homogeneity of study samples included in this review, as the majority of patients were White, male, and married. Another limitation of this systematic review is that most studies were older, with the majority conducted between 2001 and 2010, utilizing older generations of MCS devices. As a result, findings in MCS patients may not reflect the current state of device technology, potentially leading to outdated conclusions due to advancements in the field. The small number of studies included in this review is a limitation. As reported in the search strategy, a librarian worked with the team to identify relevant studies to be included in this review with the original search being conducted on July 14, 2021, and subsequently on July 17, 2023, and June 5, 2025, with the intent to find more published articles that fit eligibility criteria. Despite our efforts, we were only able to find one additional article with our June 5, 2025 search which further highlights the need for more research regarding the impact of race on HRQOL in patients who undergo advanced surgical therapies.

Lastly, a potential limitation to consider is that the systematic review included studies that characterized race as a biological variable, using an essentialism framework that categorizes populations by region of ancestry and physical traits. A constructivism framework defines race as a social construct wherein race is understood to be a composite measure with specific elements (e.g., skin color, neighborhood, region of ancestry, social status, genes, etc.) [47]. When race is operationalized as a single biological variable, it limits the ability to determine what aspects of race are most relevant to the findings and are potentially actionable. In order to gain a comprehensive understanding of the multitude of factors that influence HRQOL, it is imperative to conduct an in-depth investigation beyond the scope of race as a biological variable. By accounting for such factors, a more in-depth and nuanced understanding of factors that impact HRQOL in patients who undergo advanced cardiac surgical therapies can be gained, which ultimately may address health equity in this population of patients and improve the overall well-being of patients from diverse racial backgrounds.

## Conclusions

This systematic review evaluated peer-reviewed articles on the impact of race on HRQOL of adults with heart failure who underwent HT and/or MCS implantation (either while awaiting HT or long-term if ineligible for HT). Our findings highlight the need for future research to examine race more broadly, focusing on how factors such as healthcare access and socioeconomic determinants of health affect overall health outcomes. The world’s population is becoming increasingly multiracial; understanding how race intersects with HRQOL outcomes in patients with advanced heart failure is important to guide shared decision making when patients consider surgical treatment options and HRQOL-focused therapies upon follow-up. By synthesizing the existing literature, this systematic review identifies important gaps and highlights the potential influence of race on HRQOL following HT or MCS, demonstrating that race must be considered as a key factor in optimizing patient-centered outcomes for advanced heart failure treatment.

## Supplementary Information

Below is the link to the electronic supplementary material.

Supplementary Material 1.

## Data Availability

No datasets were generated or analysed during the current study.
