# Supplementary material for: A systematic review of the relationship between race and health-related quality of life outcomes in patients with advanced heart failure who undergo heart transplantation or mechanical circulatory support
Source: Qual Life Res. 2026 Apr 1;35(5):106. doi: 10.1007/s11136-026-04208-w (PMC13043559; doi:10.1007/s11136-026-04208-w)
Supplement: Supplementary file 1 — Supplementary Material 1. [file 11136_2026_4208_MOESM1_ESM.docx]

**A Systematic Review of the Relationship Between Race and Health-Related Quality of Life Outcomes in Patients with Advanced Heart Failure Who Undergo Heart Transplantation or Mechanical Circulatory Support**

by

Jerian Dixon-Evans, PhD, MHA^a^; 0000-0002-8946-8982

Alexis Briley, MS, RD, LDN^b^; 0000-0002-6065-9216

Jamie S. Way , PT, DPT, PhD^c^

Shondra Clay, PhD^b^; 0000-0002-8607-9463

Kathryn Mazurek, PhD, MPH^b^; 0000-0002-8350-1753

Q. Elieen Wafford, MSt, MLIS^d^; 0000-0001-5228-8219

Alyssa M. Vela, PhD^a^; 0000-0002-5308-344X

Kathleen L. Grady, PhD, RN^a^; 0000-0002-6570-5365

^a^Department of Surgery, Northwestern University, Feinberg School of Medicine, 676 North Saint Clair Street, Arkes Pavilion, suite 730, Chicago, IL 60611-3056

^b^College of Health and Human Sciences, Northern Illinois University, Wirtz Hall, 323 DeKalb, IL 60115

^c^Department of Physical Therapy & Health Science, Bradley University, Campustown 170, 1501 W. Bradley Ave., Peoria, IL 61625

^d^Feinberg School of Medicine Northwestern University, 320 E. Superior Street, Chicago, IL 60611

**Corresponding author:**

Jerian Dixon-Evans, PhD, MHA, CCRP

676 North Saint Clair Street, Arkes Pavilion, suite 730, Chicago, IL 60611-3056

Ph (618)530-5791 (cell); Email: Jerian.dixon-evans@northwestern.edu

| **Supplemental Table of Contents** | |
| --- | --- |
| Pages | |
| Table S1. Health-Related Quality of Life Measures | 3 |
| Table S2. Database Search Overview | 4 |
| Table S3. ClinicalTrials.gov – Last searched: June 5, 2025 | 4 |
| Table S4. Database(s): Ovid MEDLINE(R) and Epub Ahead of Print, In-Process, In-Data-Review & Other Non-Indexed Citations, Daily and Versions 1946 to June 4, 2025 | 5-6 |
| Table S5. The Cochrane Library | 6-9 |
| Table S6. Scopus | 9 |
| Table S7. CINAHL Plus with Full Text | 10-12 |
| Table S8. APA PsycInfo | 12-14 |
| Table S.9 PRIMSA Checklist | 15-17 |

**Supplementary Appendix Material**

| **Table S1. Health-Related Quality of Life Measures** | |
| --- | --- |
| **Measure** | **Description of Measure** |
| **Minnesota Living With Heart Failure Questionnaire** | **Disease-specific** **Health-related Quality of Life Questionnaire:**   - Heart failure-specific questionnaire with 21 items measuring HRQOL. - Three domains: physical, socio-economic and emotional/psychological aspects. Responses are Likert scaled. Total score ranges from 0 to 105, with higher scores indicating a greater impact of heart failure on QoL. |
| **Quality of life Index** | **Generic Health-related Quality of Life Questionnaire:**   - Questionnaire with 32 items measuring HRQOL. The instrument has 2 scales: satisfaction with various domains of life and importance of the domain. - Five scores are calculated: a total quality of life score and four subscale scores measuring four life domains (health and functioning, psychological/spiritual, social and economic, and family). Item scores are summed to create domain subscale scores and an overall quality of life score. Responses are based on a Likert scale (1-6): 1=very dissatisfied; 6= very satisfied. |
| **EuroQol-5 dimension-3L**  **(EQ-5D-3L)** | **Generic Health-related Quality of Life Questionnaire:**   - Overall current health status is measured using a vertical graduated (0-100) 20cm visual analog scale (VAS); VAS scores are calculated as mean+SD, 0=worst to 100=best imaginable health state. - 5 single questions (dimensions) are also assessed: self-care, mobility, usual activities, anxiety/depression, and pain/discomfort, which have a 3-level response scale: no problems, some/moderate problems, and extreme problems. |
| **Health Promoting Lifestyle Profile II** | **Health Promoting Behavior Questionnaire**   - Questionnaire with 52-items - Composed of a total scale and six subscales to measure behaviors in the theorized dimensions of health-promoting lifestyle. Six subscales: health responsibility, physical activity, nutrition, spiritual growth, interpersonal relations and stress management. Responses use a Likert scale; total score range=52 to 208. Higher total scores indicate more frequent health-promoting behaviors. |
| **Life Satisfaction Index** | **Transplant-specific Quality of Life Questionnaire:**   - A composite of 8 items measuring satisfaction with overall health. - Index scores range from 0-100. Higher scores=greater satisfaction. |
| **Kansas City Cardiomyopathy Questionnaire (KCCQ-12)** | **Disease-specific Health-related Quality of Life Questionnaire:**   - Heart Failure-specific questionnaire with 12 items measuring HRQOL. - Four domains: symptom frequency, physical limitations, social limitations, and QOL, which can be combined into an overall summary score. Responses are Likert scaled. Scale scores are transformed to 0-100. Higher scores = fewer symptoms, less physical/social limitations, better QOL, and better health status. |

| **Table S2. Database Search Overview** | | |
| --- | --- | --- |
| Database | Coverage | Results |
| Ovid MEDLINE(R) ALL 1946 to June 04, 2025 | 1946 to present | 888 |
| Cochrane Database of Systematic Reviews  Issue 6 of 12, June 2025 (Wiley) | 1995 to present | 109 |
| Cochrane Central Register of Controlled Trials  Issue 6 of 12, June 2025 (Wiley) | N/A | 140 |
| Scopus (Elsevier) | 1788 to present | 881 |
| CINAHL Plus with Full Text (EBSCOhost) | 1937 to present | 364 |
| APA PsycInfo (EBSCOhost) | 1800s to present | 173 |
| Total |  | 2555 |
| Total After De-Duplication  Removed through automation  Removed after manual review |  | 1111(7/14/21)  174 (7/17/23)  219 (6/5/25) |

We searched the databases listed above on July 14, 2021. Records from each database were exported to EndNote. Duplicates were removed using the ‘Find Duplicates’ function within EndNote and after manual review. Unique results were uploaded to Rayyan. Search strategies from each of the bibliographic databases are available below. We conducted updates to the search on July 17, 2023 and June 5, 2025.

.

**Table S3. ClinicalTrials.gov – Last searched: July 14, 2021**

URL to search results - <https://clinicaltrials.gov/ct2/results?term=mechanical+circulatory+support+OR+heart+transplant&recrs=eghim&age=2&map_cntry=US>

311 Studies found for: mechanical circulatory support OR heart transplant | Completed, Suspended, Terminated, Withdrawn, Unknown status Studies | ( Map: United States ) | Older Adult

| **Table S4. Database(s): Ovid MEDLINE(R) ALL 1946 to June 04, 2025** | | |
| --- | --- | --- |
| **#** | **Searches** | **Results** |
| 1 | exp Aged/ | 3705961 |
| 2 | exp Geriatrics/ | 32201 |
| 3 | (age or aged or elder* or frail or geriatric* or octagenarian or octogenarian or "old age" or "older adult*" or "older age" or "older female*" or "older male*" or "older man" or "older men" or "older patient*" or "older people" or "older person*" or "older population*" or "older subject*" or "older woman" or "older women" or senior or seniors or septuagenarian* or "young old" or "middle old").ti,ab. | 3934477 |
| 4 | or/1-3 | 6385712 |
| 5 | exp Heart-Assist Devices/ | 19336 |
| 6 | ("destination therapy" or "heart assist device*" or "heart assist pump*" or "mechanical left ventricular support" or "mechanical* assist* circulatory support" or "mechanical* assist* device*" or "mechanical* circulatory support" or "ventricle assist device*" or "ventricular assist device*" or LVAD or LVADs or MCS or MCSDs or VAD or VADs).ti,ab. | 54481 |
| 7 | exp Heart Transplantation/ | 41259 |
| 8 | (cardiac adj3 (graft* or transplant* or allograft*)).ti,ab. | 19054 |
| 9 | (heart adj3 (graft* or transplant* or recipient*)).ti,ab. | 38001 |
| 10 | "new heart*".ti,ab. | 713 |
| 11 | or/5-10 | 111740 |
| 12 | exp Race Factors/ | 1402 |
| 13 | exp Minority Groups/ | 20053 |
| 14 | (minority or ethnic).ti. | 37058 |
| 15 | ("communities of color" or "people of color" or minorities or race* or racial or minorities or ethnicity).ti,ab. | 308272 |
| 16 | exp African Americans/ | 78057 |
| 17 | (black or blacks).ti. | 56517 |
| 18 | ("african american*" or "black communit*").ti,ab. | 68206 |
| 19 | ((black or blacks) and (us or "u.s." or "united states" or american* or population* or ethnic* or minorit* or patient* or adult* or individual* or man or men or woman or women or participant* or racial or resident*)).ti,ab. | 134319 |
| 20 | exp Indians, North American/ | 18890 |
| 21 | ("native american*" or "north american amerind*" or "american indian*").ti,ab. | 16387 |
| 22 | ((Cherokee or Chippewa or Navajo or Sioux) and (adult* or american* or ancestry or citizen* or communit* or ethnic or indian* or indigenous or individual* or men or women or minorit* or nation* or native* or participant* or patient* or population* or racial or reservation* or resident* or tribal or tribe* or "u.s." or "united states" or "us")).ti,ab. | 1376 |
| 23 | exp Alaska Natives/ | 871 |
| 24 | ((alaska or Alaskan) and (athabascan* or ethnic or minorit* or indigenous or native* or tribal or tribe* or inuit*)).ti,ab. | 6602 |
| 25 | exp Asian Americans/ | 10126 |
| 26 | ((asian* or chinese* or filipino* or indian or indians or japanese* or korean* or philippino* or pilipino* or vietnamese*) and (american or americans)).ti,ab. | 43871 |
| 27 | exp Hispanic Americans/ | 41774 |
| 28 | (hispanic* or latino* or latina* or latinx or "puerto rican*" or "mexican american*" or "cuban american*").ti,ab. | 100621 |
| 29 | exp Oceanic Ancestry Group/ | 6494 |
| 30 | (guamanian* or "hawaiian native*" or "native hawaiian*").ti,ab. | 2624 |
| 31 | ((chamorro* or "pacific islander*" or polynesian* or samoan*) and (american or americans)).ti,ab. | 3999 |
| 32 | or/12-31 | 579203 |
| 33 | 4 and 11 and 32 | 888 |

| **Table S5. The Cochrane Library** | | |
| --- | --- | --- |
| ID | Search | Hits |
| #1 | MeSH descriptor: [Aged] explode all trees | 255415 |
| #2 | MeSH descriptor: [Geriatrics] explode all trees | 400 |
| #3 | age OR aged OR elder* OR frail OR geriatric* OR octagenarian OR octogenarian OR "old age" OR "older age" OR "older man" OR "older men" OR "older people" OR "older women" OR "older woman" OR senior OR seniors OR septuagenarian* OR "young old" OR "middle old" OR (older NEXT (adult* OR female OR male* OR person* OR population* OR subject* OR patient*)) | 868888 |
| #4 | #1 OR #2 OR #3 | 868888 |
| #5 | MeSH descriptor: [Heart-Assist Devices] explode all trees | 299 |
| #6 | "destination therapy" OR (heart assist AND device*) OR (heart assist AND pump*) OR "mechanical left ventricular support" OR (mechanical* AND assist* AND circulatory AND support) OR (mechanical* AND assist* NEXT device*) OR (mechanical* NEXT circulatory NEXT support) OR (ventricle assist NEXT device*) OR (ventricular assist NEXT device*) OR LVAD OR LVADs OR "MCS" OR MCSDs OR "VAD" OR VADs | 4159 |
| #7 | MeSH descriptor: [Heart Transplantation] explode all trees | 830 |
| #8 | (cardiac NEAR/3 (graft* OR transplant* OR allograft*)):ti,ab,kw | 955 |
| #9 | (heart NEAR/3 (graft* OR transplant* OR recipient*)):ti,ab,kw | 2892 |
| #10 | (new NEXT heart*):ti,ab,kw | 92 |
| #11 | #5 OR #6 OR #7 OR #8 OR #9 OR #10 | 7131 |
| #12 | MeSH descriptor: [Race Factors] explode all trees | 36 |
| #13 | MeSH descriptor: [Minority Groups] explode all trees | 525 |
| #14 | (minority OR ethnic):ti | 1360 |
| #15 | ("communities of color" OR "people of color" OR minorities OR race* OR racial OR minorities OR ethnicity):ti,ab,kw | 22327 |
| #16 | MeSH descriptor: [Black or African American] 2 tree(s) exploded | 0 |
| #17 | (black OR blacks):ti | 2135 |
| #18 | african american* OR black communit* | 11640 |
| #19 | ((black OR blacks) AND (us OR "u.s." OR "united states" OR american* OR population* OR ethnic* OR minorit* OR patient* OR adult* OR individual* OR man OR men OR woman OR women OR participant* OR racial OR resident*)):ti,ab,kw | 12582 |
| #20 | MeSH descriptor: [Indians, North American] 1 tree(s) exploded | 0 |
| #21 | ((native NEXT american*) OR (north AND american AND amerind*) OR (american NEXT indian*)):ti,ab,kw | 928 |
| #22 | ((cherokee OR chippewa OR navajo OR sioux) AND (adult* OR american* OR ancestry OR citizen* OR communit* OR ethnic OR indian* OR indigenous OR individual* OR men OR women OR minorit* OR nation* OR native* OR participant* OR patient* OR population* OR racial OR reservation* OR resident* OR tribal OR tribe* OR "u.s." OR "united states" OR "us")):ti,ab,kw | 91 |
| #23 | MeSH descriptor: [Alaska Natives] 2 tree(s) exploded | 0 |
| #24 | ((alaska OR alaskan) AND (athabascan* OR ethnic OR minorit* OR indigenous OR native* OR tribal OR tribe* OR inuit*)):ti,ab,kw | 360 |
| #25 | MeSH descriptor: [Asian] 2 tree(s) exploded | 0 |
| #26 | ((asian* OR chinese* OR filipino* OR indian OR indians OR japanese* OR korean* OR philippino* OR pilipino* OR vietnamese*) AND (american OR americans)):ti,ab,kw | 3517 |
| #27 | MeSH descriptor: [Hispanic or Latino] 1 tree(s) exploded | 0 |
| #28 | (hispanic* OR latino* OR latina* OR latinx OR (puerto NEXT rican*) OR (mexican NEXT american*) OR (cuban NEXT american*)):ti,ab,kw | 9149 |
| #29 | MeSH descriptor: [Native Hawaiian or Other Pacific Islander] 1 tree(s) exploded | 0 |
| #30 | (guamanian* OR (hawaiian NEXT native*) OR (native NEXT hawaiian*)):ti,ab,kw | 334 |
| #31 | ((chamorro* OR (pacific AND islander*) OR polynesian* OR samoan*) AND (american OR americans)):ti,ab,kw | 140 |
| #32 | #12 OR #13 OR #14 OR #15 OR #16 OR #17 OR #18 OR #19 OR #20 OR #21 OR #22 OR #23 OR #24 OR #25 OR #26 OR #27 OR #28 OR #29 OR #30 OR #31 | 44161 |
| #33 | #4 AND #11 AND #32 | 218 |

| **Table S6. Scopus** |
| --- |
| ( TITLE-ABS ( age OR aged OR elder* OR frail OR geriatric* OR octagenarian OR octogenarian OR "old age" OR "older adult*" OR "older age" OR "older female*" OR "older male*" OR "older man" OR "older men" OR "older patient*" OR "older people" OR "older person*" OR "older population*" OR "older subject*" OR "older woman" OR "older women" OR senior OR seniors OR septuagenarian* OR "young old" OR "middle old" ) ) AND ( TITLE-ABS ( "destination therapy" OR "heart assist device*" OR "heart assist pump*" OR "mechanical left ventricular support" OR "mechanical* assist* circulatory support" OR "mechanical* assist* device*" OR "mechanical* circulatory support" OR "ventricle assist device*" OR "ventricular assist device*" OR lvad OR lvads OR "mcs" OR mcsds OR "vad" OR vads ) OR TITLE-ABS ( cardiac W/3 ( graft* OR transplant* OR allograft* ) ) OR TITLE-ABS ( heart W/3 ( graft* OR transplant* OR recipient* ) ) OR TITLE-ABS ( "new heart*" ) ) AND ( TITLE ( minority OR ethnic ) OR TITLE-ABS ( "communities of color" OR "people of color" OR minorities OR race* OR racial OR minorities OR ethnicity ) OR TITLE ( black OR blacks ) OR TITLE-ABS ( "african american*" OR "black communit*" ) OR TITLE-ABS ( ( black OR blacks ) AND ( us OR "u.s." OR "united states" OR american* OR population* OR ethnic* OR minorit* OR patient* OR adult* OR individual* OR man OR men OR woman OR women OR participant* OR racial OR resident* ) ) OR TITLE-ABS ( "native american*" OR "north american amerind*" OR "american indian*" ) OR TITLE-ABS ( ( cherokee OR chippewa OR navajo OR sioux ) AND ( adult* OR american* OR ancestry OR citizen* OR communit* OR ethnic OR indian* OR indigenous OR individual* OR men OR women OR minorit* OR nation* OR native* OR participant* OR patient* OR population* OR racial OR reservation* OR resident* OR tribal OR tribe* OR "u.s." OR "united states" OR "us" ) ) OR TITLE-ABS ( ( alaska OR alaskan ) AND ( athabascan* OR ethnic OR minorit* OR indigenous OR native* OR tribal OR tribe* OR inuit* ) ) OR TITLE-ABS ( ( asian* OR chinese* OR filipino* OR indian OR indians OR japanese* OR korean* OR philippino* OR pilipino* OR vietnamese* ) AND ( american OR americans ) ) OR TITLE-ABS ( hispanic* OR latino* OR latina* OR latinx OR "puerto rican*" OR "mexican american*" OR "cuban american*" ) OR TITLE-ABS ( guamanian* OR "hawaiian native*" OR "native hawaiian*" ) OR TITLE-ABS ( ( chamorro* OR "pacific islander*" OR polynesian* OR samoan* ) AND ( american OR americans ) ) ) |

| **Table S7. CINAHL Plus with Full Text** |
| --- |
| # Query Limiters/Expanders Last Run Via Results  S17 S1 OR S2 OR S3 OR S4 OR S5 OR S6 OR S7 OR S8 OR S9 OR S10 OR S11 OR S12 OR S13 OR S14 OR S15 OR S16 Expanders - Apply equivalent subjects  Search modes - Proximity Interface - EBSCOhost Research Databases  Search Screen - Advanced Search  Database - CINAHL Plus with Full Text 291,273  S16 (chamorro* OR "pacific islander*" OR polynesian* OR samoan*) AND (american OR americans) Expanders - Apply equivalent subjects  Search modes - Proximity Interface - EBSCOhost Research Databases  Search Screen - Advanced Search  Database - CINAHL Plus with Full Text 2,008  S15 guamanian* OR "hawaiian native*" OR "native hawaiian*" Expanders - Apply equivalent subjects  Search modes - Proximity Interface - EBSCOhost Research Databases  Search Screen - Advanced Search  Database - CINAHL Plus with Full Text 1,209  S14 hispanic* OR latino* OR latina* OR latinx OR "puerto rican*" OR "mexican american*" OR "cuban american*" Expanders - Apply equivalent subjects  Search modes - Proximity Interface - EBSCOhost Research Databases  Search Screen - Advanced Search  Database - CINAHL Plus with Full Text 65,829  S13 (MH "Hispanic Americans") Expanders - Apply equivalent subjects  Search modes - Proximity Interface - EBSCOhost Research Databases  Search Screen - Advanced Search  Database - CINAHL Plus with Full Text 35,971  S12 (asian* OR chinese* OR filipino* OR indian OR indians OR japanese* OR korean* OR philippino* OR pilipino* OR vietnamese*) AND (american OR americans) Expanders - Apply equivalent subjects  Search modes - Proximity Interface - EBSCOhost Research Databases  Search Screen - Advanced Search  Database - CINAHL Plus with Full Text 25,428  S11 (alaska OR alaskan) AND (athabascan* OR ethnic OR minorit* OR indigenous OR native* OR tribal OR tribe* OR inuit*) Expanders - Apply equivalent subjects  Search modes - Proximity Interface - EBSCOhost Research Databases  Search Screen - Advanced Search  Database - CINAHL Plus with Full Text 3,478  S10 (cherokee OR chippewa OR navajo OR sioux) AND (adult* OR american* OR ancestry OR citizen* OR communit* OR ethnic OR indian* OR indigenous OR individual* OR men OR women OR minorit* OR nation* OR native* OR participant* OR patient* OR population* OR racial OR reservation* OR resident* OR tribal OR tribe* OR "u.s." OR "united states" OR "us") Expanders - Apply equivalent subjects  Search modes - Proximity Interface - EBSCOhost Research Databases  Search Screen - Advanced Search  Database - CINAHL Plus with Full Text 578  S9 "native american*" OR "north american amerind*" OR "american indian*" Expanders - Apply equivalent subjects  Search modes - Proximity Interface - EBSCOhost Research Databases  Search Screen - Advanced Search  Database - CINAHL Plus with Full Text 12,811  S8 (MH "Native Americans+") Expanders - Apply equivalent subjects  Search modes - Proximity Interface - EBSCOhost Research Databases  Search Screen - Advanced Search  Database - CINAHL Plus with Full Text 9,580  S7 (black OR blacks) AND (us OR "u.s." OR "united states" OR american* OR population* OR ethnic* OR minorit* OR patient* OR adult* OR individual* OR man OR men OR woman OR women OR participant* OR racial OR resident*) Expanders - Apply equivalent subjects  Search modes - Proximity Interface - EBSCOhost Research Databases  Search Screen - Advanced Search  Database - CINAHL Plus with Full Text 70,071  S6 "african american*" OR "black communit*" Expanders - Apply equivalent subjects  Search modes - Proximity Interface - EBSCOhost Research Databases  Search Screen - Advanced Search  Database - CINAHL Plus with Full Text 39,540  S5 TI black OR blacks Expanders - Apply equivalent subjects  Search modes - Proximity Interface - EBSCOhost Research Databases  Search Screen - Advanced Search  Database - CINAHL Plus with Full Text 80,738  S4 "communities of color" OR "people of color" OR minorities OR race* OR racial OR minorities OR ethnicity Expanders - Apply equivalent subjects  Search modes - Proximity Interface - EBSCOhost Research Databases  Search Screen - Advanced Search  Database - CINAHL Plus with Full Text 170,917  S3 TI minority OR ethnic Expanders - Apply equivalent subjects  Search modes - Proximity Interface - EBSCOhost Research Databases  Search Screen - Advanced Search  Database - CINAHL Plus with Full Text 77,841  S2 (MH "Minority Groups") Expanders - Apply equivalent subjects  Search modes - Proximity Interface - EBSCOhost Research Databases  Search Screen - Advanced Search  Database - CINAHL Plus with Full Text 15,893  S1 (MH "Race Factors") Expanders - Apply equivalent subjects  Search modes - Proximity Interface - EBSCOhost Research Databases  Search Screen - Advanced Search  Database - CINAHL Plus with Full Text 35,510 |

| **Table S8. APA PsycInfo** |
| --- |
| # Query Limiters/Expanders Last Run Via Results  S20 S1 AND S6 AND S19 Expanders - Apply equivalent subjects  Search modes - Proximity Interface - EBSCOhost Research Databases  Search Screen - Advanced Search  Database - APA PsycInfo 173  S19 S7 OR S8 OR S9 OR S10 OR S11 OR S12 OR S13 OR S14 OR S15 OR S16 OR S17 OR S18 Expanders - Apply equivalent subjects  Search modes - Proximity Interface - EBSCOhost Research Databases  Search Screen - Advanced Search  Database - APA PsycInfo 394,540  S18 (chamorro* OR "pacific islander*" OR polynesian* OR samoan*) AND (american OR americans) Expanders - Apply equivalent subjects  Search modes - Proximity Interface - EBSCOhost Research Databases  Search Screen - Advanced Search  Database - APA PsycInfo 2,153  S17 guamanian* OR "hawaiian native*" OR "native hawaiian*" Expanders - Apply equivalent subjects  Search modes - Proximity Interface - EBSCOhost Research Databases  Search Screen - Advanced Search  Database - APA PsycInfo 1,188  S16 hispanic* OR latino* OR latina* OR latinx OR "puerto rican*" OR "mexican american*" OR "cuban american*" Expanders - Apply equivalent subjects  Search modes - Proximity Interface - EBSCOhost Research Databases  Search Screen - Advanced Search  Database - APA PsycInfo 80,864  S15 (asian* OR chinese* OR filipino* OR indian OR indians OR japanese* OR korean* OR philippino* OR pilipino* OR vietnamese*) AND (american OR americans) Expanders - Apply equivalent subjects  Search modes - Proximity Interface - EBSCOhost Research Databases  Search Screen - Advanced Search  Database - APA PsycInfo 42,158  S14 (alaska OR alaskan) AND (athabascan* OR ethnic OR minorit* OR indigenous OR native* OR tribal OR tribe* OR inuit*) Expanders - Apply equivalent subjects  Search modes - Proximity Interface - EBSCOhost Research Databases  Search Screen - Advanced Search  Database - APA PsycInfo 3,646  S13 (cherokee OR chippewa OR navajo OR sioux) AND (adult* OR american* OR ancestry OR citizen* OR communit* OR ethnic OR indian* OR indigenous OR individual* OR men OR women OR minorit* OR nation* OR native* OR participant* OR patient* OR population* OR racial OR reservation* OR resident* OR tribal OR tribe* OR "u.s." OR "united states" OR "us") Expanders - Apply equivalent subjects  Search modes - Proximity Interface - EBSCOhost Research Databases  Search Screen - Advanced Search  Database - APA PsycInfo 2,016  S12 "native american*" OR "north american amerind*" OR "american indian*" Expanders - Apply equivalent subjects  Search modes - Proximity Interface - EBSCOhost Research Databases  Search Screen - Advanced Search  Database - APA PsycInfo 13,177  S11 (black OR blacks) AND (us OR "u.s." OR "united states" OR american* OR population* OR ethnic* OR minorit* OR patient* OR adult* OR individual* OR man OR men OR woman OR women OR participant* OR racial OR resident*) Expanders - Apply equivalent subjects  Search modes - Proximity Interface - EBSCOhost Research Databases  Search Screen - Advanced Search  Database - APA PsycInfo 111,572  S10 "african american*" OR "black communit*" Expanders - Apply equivalent subjects  Search modes - Proximity Interface - EBSCOhost Research Databases  Search Screen - Advanced Search  Database - APA PsycInfo 65,053  S9 TI black OR blacks Expanders - Apply equivalent subjects  Search modes - Proximity Interface - EBSCOhost Research Databases  Search Screen - Advanced Search  Database - APA PsycInfo 121,639  S8 "communities of color" OR "people of color" OR minorities OR race* OR racial OR minorities OR ethnicity Expanders - Apply equivalent subjects  Search modes - Proximity Interface - EBSCOhost Research Databases  Search Screen - Advanced Search  Database - APA PsycInfo 247,735  S7 DE "Alaska Natives" OR DE "American Indians" OR DE "Hawaii Natives" OR DE "Inuit" OR DE "Pacific Islanders" OR DE "Racial and Ethnic Groups" OR DE "Asians" OR DE "Blacks" OR DE "Indigenous Populations" OR DE "Latinos/Latinas" OR DE "Minority Groups" Expanders - Apply equivalent subjects  Search modes - Proximity Interface - EBSCOhost Research Databases  Search Screen - Advanced Search  Database - APA PsycInfo 92,231  S6 S2 OR S3 OR S4 OR S5 Expanders - Apply equivalent subjects  Search modes - Proximity Interface - EBSCOhost Research Databases  Search Screen - Advanced Search  Database - APA PsycInfo 4,538  S5 "new heart*" Expanders - Apply equivalent subjects  Search modes - Proximity Interface - EBSCOhost Research Databases  Search Screen - Advanced Search  Database - APA PsycInfo 39  S4 heart N3 (graft* OR transplant* OR recipient*) Expanders - Apply equivalent subjects  Search modes - Proximity Interface - EBSCOhost Research Databases  Search Screen - Advanced Search  Database - APA PsycInfo 643  S3 cardiac N3 (graft* OR transplant* OR allograft*) Expanders - Apply equivalent subjects  Search modes - Proximity Interface - EBSCOhost Research Databases  Search Screen - Advanced Search  Database - APA PsycInfo 191  S2 "destination therapy" OR "heart assist device*" OR "heart assist pump*" OR "mechanical left ventricular support" OR "mechanical* assist* circulatory support" OR "mechanical* assist* device*" OR "mechanical* circulatory support" OR "ventricle assist device*" OR "ventricular assist device*" OR LVAD OR LVADs OR "MCS" OR MCSDs OR "VAD" OR VADs Expanders - Apply equivalent subjects  Search modes - Proximity Interface - EBSCOhost Research Databases  Search Screen - Advanced Search  Database - APA PsycInfo 3,867  S1 age OR aged OR elder* OR frail OR geriatric* OR octagenarian OR octogenarian OR "old age" OR "older adult*" OR "older age" OR "older female*" OR "older male*" OR "older man" OR "older men" OR "older patient*" OR "older people" OR "older person*" OR "older population*" OR "older subject*" OR "older woman" OR "older women" OR senior OR seniors OR septuagenarian* OR "young old" OR "middle old" Expanders - Apply equivalent subjects  Search modes - Proximity Interface - EBSCOhost Research Databases  Search Screen - Advanced Search  Database - APA PsycInfo 1,334,275 |

| **Table S9. PRISMA 2020 Checklist** | | |  |
| --- | --- | --- | --- |
| **Section and Topic** | **Item #** | **Checklist item** | **Location where item is reported** |
| **TITLE** | | |  |
| Title | 1 | Identify the report as a systematic review. | 1 |
| **ABSTRACT** | | |  |
| Abstract | 2 | See the PRISMA 2020 for Abstracts checklist. | 2 |
| **INTRODUCTION** | | |  |
| Rationale | 3 | Describe the rationale for the review in the context of existing knowledge. | 4-5 |
| Objectives | 4 | Provide an explicit statement of the objective(s) or question(s) the review addresses. | 5 |
| **METHODS** | | |  |
| Eligibility criteria | 5 | Specify the inclusion and exclusion criteria for the review and how studies were grouped for the syntheses. | 6; 25  (Table 1) |
| Information sources | 6 | Specify all databases, registers, websites, organisations, reference lists and other sources searched or consulted to identify studies. Specify the date when each source was last searched or consulted. | 5-6; (Supplemental Table S2) |
| Search strategy | 7 | Present the full search strategies for all databases, registers and websites, including any filters and limits used. | Supplemental Tables S2-S8) |
| Selection process | 8 | Specify the methods used to decide whether a study met the inclusion criteria of the review, including how many reviewers screened each record and each report retrieved, whether they worked independently, and if applicable, details of automation tools used in the process. | 6-7 |
| Data collection process | 9 | Specify the methods used to collect data from reports, including how many reviewers collected data from each report, whether they worked independently, any processes for obtaining or confirming data from study investigators, and if applicable, details of automation tools used in the process. | 8 |
| Data items | 10a | List and define all outcomes for which data were sought. Specify whether all results that were compatible with each outcome domain in each study were sought (e.g. for all measures, time points, analyses), and if not, the methods used to decide which results to collect. | 8 |
|  | 10b | List and define all other variables for which data were sought (e.g. participant and intervention characteristics, funding sources). Describe any assumptions made about any missing or unclear information. | 8 |
| Study risk of bias assessment | 11 | Specify the methods used to assess risk of bias in the included studies, including details of the tool(s) used, how many reviewers assessed each study and whether they worked independently, and if applicable, details of automation tools used in the process. | 8, 10-11 |
| Effect measures | 12 | Specify for each outcome the effect measure(s) (e.g. risk ratio, mean difference) used in the synthesis or presentation of results. | ----- |
| Synthesis methods | 13a | Describe the processes used to decide which studies were eligible for each synthesis (e.g. tabulating the study intervention characteristics and comparing against the planned groups for each synthesis (item #5)). | 9-10 |
|  | 13b | Describe any methods required to prepare the data for presentation or synthesis, such as handling of missing summary statistics, or data conversions. | ----- |
|  | 13c | Describe any methods used to tabulate or visually display results of individual studies and syntheses. | ----- |
|  | 13d | Describe any methods used to synthesize results and provide a rationale for the choice(s). If meta-analysis was performed, describe the model(s), method(s) to identify the presence and extent of statistical heterogeneity, and software package(s) used. | 8-9 |
|  | 13e | Describe any methods used to explore possible causes of heterogeneity among study results (e.g. subgroup analysis, meta-regression). | ---- |
|  | 13f | Describe any sensitivity analyses conducted to assess robustness of the synthesized results. | ----- |
| Reporting bias assessment | 14 | Describe any methods used to assess risk of bias due to missing results in a synthesis (arising from reporting biases). | ------ |
| Certainty assessment | 15 | Describe any methods used to assess certainty (or confidence) in the body of evidence for an outcome. | ------- |
| **RESULTS** | | |  |
| Study selection | 16a | Describe the results of the search and selection process, from the number of records identified in the search to the number of studies included in the review, ideally using a flow diagram. | 7 |
|  | 16b | Cite studies that might appear to meet the inclusion criteria, but which were excluded, and explain why they were excluded. | 7 |
| Study characteristics | 17 | Cite each included study and present its characteristics. | 26-31 (Table 2) |
| Risk of bias in studies | 18 | Present assessments of risk of bias for each included study. | 6 |
| Results of individual studies | 19 | For all outcomes, present, for each study: (a) summary statistics for each group (where appropriate) and (b) an effect estimate and its precision (e.g. confidence/credible interval), ideally using structured tables or plots. | 6 |
| Results of syntheses | 20a | For each synthesis, briefly summarise the characteristics and risk of bias among contributing studies. | 32-34 |
|  | 20b | Present results of all statistical syntheses conducted. If meta-analysis was done, present for each the summary estimate and its precision (e.g. confidence/credible interval) and measures of statistical heterogeneity. If comparing groups, describe the direction of the effect. | 9-12 |
|  | 20c | Present results of all investigations of possible causes of heterogeneity among study results. | ------ |
|  | 20d | Present results of all sensitivity analyses conducted to assess the robustness of the synthesized results. | ------ |
| Reporting biases | 21 | Present assessments of risk of bias due to missing results (arising from reporting biases) for each synthesis assessed. | ------ |
| Certainty of evidence | 22 | Present assessments of certainty (or confidence) in the body of evidence for each outcome assessed. | ------ |
| **DISCUSSION** | | |  |
| Discussion | 23a | Provide a general interpretation of the results in the context of other evidence. | 12-14 |
|  | 23b | Discuss any limitations of the evidence included in the review. | 15-16 |
|  | 23c | Discuss any limitations of the review processes used. | 15-16 |
|  | 23d | Discuss implications of the results for practice, policy, and future research. | 16 |
| **OTHER INFORMATION** | | |  |
| Registration and protocol | 24a | Provide registration information for the review, including register name and registration number, or state that the review was not registered. | 5 |
|  | 24b | Indicate where the review protocol can be accessed, or state that a protocol was not prepared. | 5 |
|  | 24c | Describe and explain any amendments to information provided at registration or in the protocol. | ------ |
| Support | 25 | Describe sources of financial or non-financial support for the review, and the role of the funders or sponsors in the review. | 24 |
| Competing interests | 26 | Declare any competing interests of review authors. | 24 |
| Availability of data, code and other materials | 27 | Report which of the following are publicly available and where they can be found: template data collection forms; data extracted from included studies; data used for all analyses; analytic code; any other materials used in the review. | Supplemental Tables S2-S8 |

From: Page MJ, McKenzie JE, Bossuyt PM, Boutron I, Hoffmann TC, Mulrow CD, et al. The PRISMA 2020 statement: an updated guideline for reporting systematic reviews. BMJ 2021;372:n71. doi: 10.1136/bmj.n71. This work is licensed under CC BY 4.0. To view a copy of this license, visit https://creativecommons.org/licenses/by/4.0/
